# Prevalence of Type 2 Diabetes, Overweight, Obesity, and Metabolic Syndrome in Adults in Bogotá, Colombia, 2022–2023: A Cross‑Sectional Population Survey

**DOI:** 10.5334/aogh.4539

**Published:** 2024-11-11

**Authors:** Juan M. Arteaga, Catalina Latorre-Santos, Milciades Ibáñez-Pinilla, Magnolia del Pilar Ballesteros-Cabrera, Leyvi Y. Barón, Sergio A. Velosa, Carlos E. Trillos, Juan J. Duque, Andrea Holguín, Javier H. Eslava-Schmalbach

**Affiliations:** 1 School of Medicine, Universidad Nacional de Colombia, Chief of Endocrinology Department, Hospital Universitario Nacional de Colombia, Bogotá, Colombia; 2 School of Medicine and Health Sciences, Public Health Research Group, Universidad del Rosario, Bogotá, Colombia; 3 School of Medicine and Health Sciences, Universidad del Rosario, Bogotá, Colombia; 4 Department of Psychology Associate Professor and Director of the Lifestyle and Human Development Research Group, Universidad Nacional de Colombia, Bogotá, Colombia; 5 Vice Dean of Research and Extension Office, School of Medicine, Universidad Nacional de Colombia, Bogotá, Colombia; 6 Health Equity Research Group, School of Medicine, Universidad Nacional de Colombia; 7 Hospital Universitario Nacional de Colombia, Bogotá, Colombia; 8 Endocrinologist, Universidad Nacional de Colombia, Bogotá, Colombia; 9 Endocrinology Fellow, Universidad Nacional de Colombia, Bogotá, Colombia

**Keywords:** diabetes mellitus, epidemiology, overweight/obesity, metabolic syndrome, latin america, colombia

## Abstract

*Objective:* To establish the prevalence of type 2 diabetes, overweight/obesity, and metabolic syndrome in individuals aged >18 years in Bogotá, Colombia and the variables associated with diabetes prevalence.

*Research Design and Methods:* This was a cross‑sectional population survey with a representative, probabilistic sample of Bogotá, Colombia collected between 2022 and 2023. The final sample size included 2,860 households, distributed among 19 localities of Bogotá. Clinical laboratory samples were taken from randomly selected individuals (*n* = 1,070). Data on the Adult Treatment Panel III (ATP III) and Latin American Diabetes Association (ALAD) criteria for metabolic syndrome were collected, including physical measurements.

*Results:* The prevalence of type 2 diabetes in Bogotá was 11.0% (95% confidence interval [CI], 9.0–13.5%). According to the ATP III and ALAD criteria, the prevalence proportions of metabolic syndrome were 33.9% (95% CI, 29.5–38.6) and 29.3% (95% CI, 26.1–32.7), respectively. The age of ≥55 years, abdominal obesity, hypertriglyceridemia, and noneducational level had higher adjusted prevalence ratios (APRs) of diabetes. The APRs of metabolic syndrome were higher in adults with a low education level (LEL) and female sex, with the ATP III and ALAD criteria, and noninsured adults or those with unknown affiliation with the healthcare system, with the ATP III criteria.

*Conclusions:* We found a higher prevalence of type 2 diabetes in adults in Bogotá than expected in previous studies. Intervention from public policy should be requested, especially in those of lowest socioeconomic and education levels, to avoid a future increase in this prevalence. Studies on other Colombian cities are required.

## Article Highlights

*a. Why did we undertake this study?*

The city of Bogotá did not have a study on the prevalence of diabetes, metabolic syndrome, or their risk factors that would facilitate the implementation of public policies for timely diagnosis and proper diabetes control.

*b. What is the specific question(s) we wanted to answer?*

What is the prevalence of type 2 diabetes and metabolic syndrome in Bogotá?

*c. What did we find?*

The estimated prevalence of type 2 diabetes in individuals aged ≥18 years in Bogotá was 11.0% (95% confidence interval, 9.0–13.5%), and the prevalence of metabolic syndrome varied according to the Adult Treatment Panel III (33.9%) and Latin American Diabetes Association (29.3%) criteria.

*d. What are the implications of our findings?*

The prevalence proportions of type 2 diabetes, overweight/obesity, and metabolic syndrome are higher than estimated, which should encourage the authorities, health personnel, and society in general to change their environments, policies, and habits to take action and prevent this increasing tendency.

## Background

Diabetes is the sixth leading cause of mortality worldwide [1]. Adults with diabetes are two to four times more likely to die from heart disease than those without diabetes [2]. Proper diabetes control can decrease the associated risks of cardiovascular and kidney diseases and dementia [2].

The prevalence of adults with diabetes worldwide increased from 4.7% in 1980 to 8.5% in 2014 [1], which corresponded to an increase in the number of people with diabetes from 108 million in 1980 to 422 million in 2014 [3] and to 537 million adults (20–79 years) in 2021 [4] The prevalence has increased more rapidly in low‑ and middle‑income countries than in high‑income countries [3].

In addition to cardiovascular and cerebrovascular diseases, diabetes affects target organs, leading to blindness, kidney failure, neuropathy that can cause amputations, and associated healthcare costs [5, 6].

A 5% increase in premature mortality due to diabetes was noted between 2000 and 2016. In 2016, 1.6 million deaths were directly attributed to diabetes. In 2012, 2 million deaths were attributed to high glucose levels [7].

Timely diagnosis and proper control of diabetes can prevent or delay the onset of chronic complications. Interventions such as a healthy diet, regular physical activity, weight control, blood pressure management, and appropriate and timely medication are associated with an effective reduction in adverse outcomes [2].

Among the conditions that predispose to the development of type 2 diabetes, the so‑called metabolic syndrome stands out. This diagnostic category was proposed in the 1980s by researcher Gerald Reaven, who initially called it “syndrome X” [8].

The components of metabolic syndrome include abdominal obesity, hypertension, hypertriglyceridemia, low high‑density lipoprotein (HDL) cholesterol levels, and fasting dysglycemia or carbohydrate intolerance [9].

A healthy diet, regular physical activity and weight control can delay or prevent the onset of diabetes in individuals at risk [2]. Conversely, a high‑sugar and high‑calorie diet, overweight and obesity, physical inactivity associated with lifestyle, and genetic factors increase the likelihood of developing diabetes [2].

Colombia is an upper‑middle‑income country, with a segmented health care system, where not‑working patients are covered with government support (subsidized regimen), while the others, dependent and independent workers, are included within the health care system with workers’ or workers’ and employers’ contributions (contributive regimen) [10, 11]. The monetary poverty level in 2023 (people living with less than US$ 100, 6‑monthly (COP$ 435.375 with a change rate of COP$ 4,325.95 per dollar) was 33% and for Bogotá, the capital, with around 8 million people, the monetary poverty level was 23.7% [12]. From the same source, income inequality measured by Gini coefficient was 0.53 and 0.52 for Colombia and Bogotá, respectively. Fifteen percent of the entire Colombian population and 52% of residents in the five largest cities live in Bogotá [12]. The rapid population growth over the past three decades, partly due to forced displacement from various regions, has led to significant changes in the city’s lifestyle, transportation methods, work modalities, and eating habits [13].

Although some organizations such as the International Diabetes Federation (IDF), as indicated in 2017 [14], reported that the prevalence of diabetes mellitus in Colombia was 8.2% and projected that it would reach 10% by 2045 based on our clinical and epidemiological investigations, we perceive that these prevalence rates could be significantly higher. Consequently, it is imperative to conduct field research to ascertain the accurate figures within our city.

In the future, it will be very important to explore other variables such as the social determinants of the disease as well as the vulnerabilities of the population affected by this condition.

In Colombia, the prevalence of diabetes in adults is approximately 8%, making it a public health priority. The goal of reducing diabetes‑related mortality by 25% by 2021, as well as minimizing the progression to chronic kidney disease, has been proposed in the Decennial Public Health Plan [3, 7].

In the most recent study in Colombia in 2015, a review of available literature reported a diabetes prevalence of 8.5% in the adult population [15]. In the report on high‑cost diseases, which included all‑aged individuals, but only included those diagnosed within the healthcare system, the estimated age‑adjusted prevalence for 2022 was between 3.89% and 2.17% for the contributory and subsidized regimens, respectively [16]. This shows a differential gap between the affiliates of both regimens in the proportion of undiagnosed patients with diabetes, and therefore, uncontrolled, which are approximately 4.8% for the contributory regimen and 6.4% for the subsidized regimen.

The “Cities Changing Diabetes” strategy has been performed in several major cities worldwide to improve the diagnosis and timely management of diabetes [17, 18]. Bogotá was included as part of this global strategy, with the support of the District Health Secretariat.

This study aimed to establish the prevalence of type 2 diabetes and metabolic syndrome in individuals aged ≥18 years in Bogotá, Colombia and the variables associated with diabetes prevalence.

## Research Design and Methods

This is a cross‑sectional study carried out between 2022 and 2023, with a representative sample of Bogotá (Colombia).

The population under study was made up of men and women over 18 years of age, residing in households in Bogotá between 2022 and 2023, identified with the sampling frame (database and cartography) of the year 2018 population census of the National Administrative Department of Statistics (DANE), and made up of *N*1 = 43,333 blocks, *N*2 = 2,334,795 dwellings, *N*3 = 2,509,581 households, and a total of *N*4 = 6,277,951 people from Bogotá households.

The sample was probabilistic without replacement with a multistage design, randomly stratified by clusters. The sample was probabilistic since each sector, census section, block, segment, dwelling, household, and person had an a priori probability >0 of being selected; it was stratified by the 19 localities of the city (excluding one, namely Sumapaz, due to its rural predominance) and socioeconomic strata with a multistage design where the primary sampling unit (UPM) was the census sectors, the secondary sampling unit (USM) was the census sections, and the tertiary sampling unit (UTM) was the segments (ten households). In the fourth stage (UCM), properties were selected, in the fifth stage (UQM), dwellings were selected, in the sixth stage (USEM), households were selected, and in the seventh stage (USEPM), people were selected. The first six sampling units formed the clusters and the selection was random in all stages.

The sample size was obtained by taking into account the variability of the prevalence of type 2 diabetes in similar Colombian population studies, which varied between 7.1% and 8.5% [15], by using absolute precision of ≤1% and relative standard error of ≤15%, 95% reliability, and cluster design effect of 1.5. The sample size varied between 2,766 and 3,350 households, with a 10% adjustment for losses. The final sample was 2,860 households, distributed in the 19 localities of Bogotá, and 1,070 laboratory tests were taken from individuals in randomly selected households.

The information was collected with a structured questionnaire applied face to face with the randomly selected person from the household. A pilot test was previously carried out in different socioeconomic strata, sexes, and age groups. The final questionnaire was systematized into a Research Electronic Data Capture (REDCap) database provided by Vanderbilt University [19], located at the School of Medicine of the Universidad Nacional de Colombia, using tablets and mobile phones, which stored the data on a private server to ensure confidentiality [19]. In places with connectivity problems or for security reasons, the questionnaires were collected in physical form. The information collection and systematization team was made up of nutrition and nursing professionals supported by technical nursing staff and a field coordinator. This field work team was trained in research knowledge, data collection instruments, standardization of anthropometric and clinical measurements, random selection of homes and individuals, and data collection and systematization in homes of individuals.

The variables of the questionnaire were made up of sociodemographic data and nutritional, clinical, and paraclinical habits. The main variables (type 2 diabetes and metabolic syndrome) were defined according to the Adult Treatment Panel III (ATP III) [20], the American Heart Association [21], National Heart, Lung, and Blood Institute [22], and the Latin American Diabetes Association (ALAD) [23].

The diagnosis of type 2 diabetes was done with a hemoglobin A1c (HbA1c) level of > 6.5% (48 mmol/mol) [24] and self‑reported prevalent cases. Metabolic syndrome was defined on the basis of the following criteria: abdominal obesity, elevated triglyceride level, low HDL cholesterol level, arterial hypertension, and dysglycemia. According to the ATP III criteria, metabolic syndrome exists when at least three of the five criteria are met [20]. According to ALAD, metabolic syndrome is diagnosed when abdominal obesity is present, along with two of the other four criteria [23].

Abdominal obesity was defined as a waist circumference of >94 cm for men or >88 cm for women. Overweight was classified as a body mass index (BMI) between 25.0 and 29.9 kg/m^2^ and obesity was classified as a BMI of ≥30.0 kg/m^2^. Obesity was classified as: obesity class I (30.0–34.9 kg/m^2^), obesity class II (35.0–39.9 kg/m^2^), and obesity class III (≥40.0 kg/m^2^). Metabolic syndrome was defined as high triglyceride level (>150 mg/dl), or being under specific hypolipidemic treatment, or low HDL cholesterol level (<40 mg/dl in men or <50 mg/dl in women). A normal high blood pressure (BP) was defined as a systolic BP of >130 mmHg or diastolic BP of >85 mmHg. Hypertension was defined as a systolic BP of >140 mmHg or diastolic BP of >90 mmHg or being under antihypertensive treatment [25, 26].

### Cross‑sectional study analysis

Data systematization involved creating the study questionnaire’s database structure for input in REDCap, and data were cleaned using SPSS version 25.0 and STATA version 15.0. The processing of information was performed using the complex sample data analysis module of SPSS, following the multistage stratified probabilistic design of the study. The sample’s precision was assessed by estimating the standard errors and relative standard errors of sampling and evaluating the precision of estimators according to Canada’s quality parameters, according to the following classifications [27]:

- Quality A: 0–15%, good precision in estimates.
- Quality B: 15.1–30%, acceptable precision.
- Quality C: 30.1–50%, estimates should be interpreted with caution.
- Quality D: >50%, non‑publishable estimates and minimum degree of precision.

Estimates were extrapolated or inferred to the population using weighting based on the DANE’s sampling frame and projections for 2023, taking into account the multistage stratified probabilistic design.

Bivariate analysis was conducted to examine the association between clinical and socioeconomic factors and the prevalence of type 2 diabetes in Bogotá, using Pearson’s chi‑squared test of independence and the likelihood ratio test with asymptotic and exact stratified multistage complex sample designs and prevalence ratios and their respective 95% confidence intervals (CIs).

For numerical scale variables, normality was evaluated using the Kolmogorov–Smirnov test with Lilliefors corrections. Parametric or nonparametric analysis of variance was used depending on the fulfillment of assumptions of normality and homogeneity of variances (Bartlett–Box test).

Descriptive statistics were used, with qualitative variables presented as absolute and relative frequencies expressed in percentages and numerical variables with measures of central tendency (mean and median), dispersion (range, interquartile range, and standard deviation), and variation or homogeneity (relative standard error).

Crude and adjusted analyses were performed using negative log‑binomial regression models for stratified multistage sampling [28–31]. These multivariate models were used to identify factors associated with type 2 diabetes (according to the ATP III and ALAD criteria) [20] and type 2 diabetes risk [32] while controlling for confounding variables among clinical and sociodemographic variables in the model.

The multicollinearity was evaluated between socioeconomic variables, and the education level was included in the model.

Statistical analyses were performed using Statistical packages SPSS version 25.0, STATA 16.1, and R (v.1.1419). Statistical tests were evaluated at a significance level of 5% (*p* < 0.05).

### Ethical aspects

This study followed the guidelines of “Good Clinical Practices,” was considered minimal risk according to the Colombian 8430 resolution of 1993 issued by the Ministry of Health, and respected the confidentiality of all participants in data collection. The database was structured in REDCap, which protected the confidentiality of sensitive information through data encryption. All participants signed a written informed consent, explaining the study’s purpose, sampling, physical measurements, and possibility of a second visit in case of a positive result to assess risk factors. Participants were informed on‑site about their risk level and received instructions on appropriate actions based on their reported risk level. A copy of their laboratory test results was provided to each participant, and they were advised to seek necessary assistance from their healthcare insurance company in case of a positive diabetes diagnosis based on the laboratory results. The same procedure was followed for those with high BP values during the survey.

This study was approved by the Ethics Committee at Bogotá’s District Health Secretariat on 17 September 2021 (code, CIE SDSCTI20210012; response number, 2021EE90468).

### Data and resource availability

The data that support the findings of this study are available on request.

## Results

In this study of the prevalence of type 2 diabetes and metabolic syndrome in Bogotá, a representative sample of 2,860 households was included among the 19 localities of Bogotá between 2022 and 2023, in which 65.3% (*n* = 1,856) were female (Supplementary Table S1).

In the households of the representative sample in Bogotá, over 93% had access to electricity, water supply, natural gas, sewerage, and garbage collection services (Supplementary Table S2). The most frequent socioeconomic strata were low and medium low, representing 81.7% of the sample, and households were more often occupied for rent, followed by owned, with nine out of ten households having their own kitchen (Supplementary Table S2).

Regarding the study participants, the most frequent sex was female, and the most common age groups were 60–69 years and 50–59 years (Supplementary Table S1). The most frequent education level was completed middle education, and the most common occupation was working with verbal and written contracts. The most frequent income was the minimum wage, which applied to approximately 50% of the participants (Supplementary Table S1).

Eighteen percent of individuals surveyed reported contributing to a current pension fund and family compensation fund (Supplementary Table S3). Participants mentioned having performed unpaid activities last week, other unpaid activities (38.1%), taking care of or attending to children (10.8%), performing tasks in other households or institutions (5.6%), and taking care of sick, elderly, and/or disabled people (5.5%), among others (Supplementary Table S3).

### Clinical characteristics

Individuals surveyed within households reported the following diseases in order of frequency: high BP, type 2 diabetes, high cholesterol level, gastroesophageal reflux, and heart disease (Table 1).

**Table 1:** Prevalent cases of self‑reported diseases in individuals aged ≥18 years in Bogotá, 2022–2023.

PREVALENCE OF SELF‑REPORTED DISEASE	FREQUENCY	PERCENTAGE
**Total population**	5,410,337	100.0%
**Heart disease**	274,364	5.1%
**High blood pressure**	1,239,532	22.9%
**High cholesterol level**	550,438	10.2%
**Type 2 diabetes**	406,322	7.5%
**Gastroesophageal reflux**	32,265	6.0%
**Gout**	3,038	0.6%
**Other diseases**	2,799,972	51.8%

*Source*: authors.

### Prevalence of type 2 diabetes in Bogotá, Colombia, 2022–2023

The estimated prevalence of type 2 diabetes in individuals aged ≥18 years in Bogotá was 11.0% (95% CI, 9.0%–13.5%; relative standard error, 10.5%) (Table 2).

**Table 2:** Prevalence of diabetes, metabolic syndrome, abdominal obesity and other related conditions in the population aged ≥in 18 years in Bogotá (Colombia) 2022–2023.

PREVALENCE	ESTIMATION	STANDARD ERROR	95% CONFIDENCE INTERVAL		RELATIVE STANDARD ERROR	DESIGN EFFECT
			LOWER	UPPER		
**Diabetes**	11.0%	1.20%	9.0%	13.5%	0.105	1.446
**Metabolic syndrome, ATP III**	33.9%	2.30%	29.5%	38.6%	0.068	2.502
**Metabolic syndrome, ALAD**	29.3%	1.70%	26.1%	32.7%	0.057	1.394
**Abdominal obesity**	47.8%	1.30%	45.2%	50.3%	0.027	1.847
**High triglyceride level**	44.1%	2.30%	39.6%	48.7%	0.053	2.361
**Low HDL cholesterol level**	42.6%	2.10%	38.6%	46.8%	0.049	1.914
**Hypertension**	28.3%	1.00%	26.3%	30.4%	0.037	1.519
**Abnormal fasting glucose level**	11.2%	1.30%	8.9%	14.0%	0.116	1.809

*Source*: authors.

*Note*: ATP III, Adult Treatment Panel III; ALAD, Latin American Diabetes Association; HDL, high‑density lipoprotein.

The prevalence of metabolic syndrome varied according to the ATP III (33.9%) or ALAD (29.3%) criteria (Table 2). The prevalence of each of the criteria used in the definition of metabolic syndrome is presented in Table 2. The estimated prevalence of metabolic syndrome using the ATP III criteria was higher than the prevalence estimated according to the ALAD criteria (Table 2). The prevalence of abdominal obesity, high triglyceride level, and low HDL cholesterol level was >42% in all these cases (Table 2).

The variables associated with the prevalence of diabetes are presented in Table 3, along with their respective crude prevalence ratios and adjusted prevalence ratios (APRs). The unadjusted prevalence ratios and APRs of diabetes were higher among individuals aged >55 years, and those who had high triglyceride levels, abdominal obesity, and no education (Table 3).

**Table 3:** Prevalence of type 2 diabetes and metabolic syndrome in the population aged ≥18 years in Bogotá, Colombia, 2022–2023.

	UNADJUSTED			ADJUSTED		
	PR	95% CI	*P* VALUE	PR	95% CI	*P* VALUE
**Female**	1.14	(0.80–1.62)	0.481	1.39	(0.96–2.04)	0.080
**Age, years**						
**<45 (ref.)**	1			1		
**45–54.9**	2.78	(1.06–7.29)	0.038	2.58	(0.99–6.74)	0.053
**55–64.9**	5.48	(2.29–13.2)	<0.001	4.46	(1.79–11.15)	0.001
**≥65**	10.47	(4.43–24.8)	<0.001	5.89	(2.42–14.30)	<0.001
**Abdominal obesity**	3.30	(2.32–4.68)	<0.001	2.00	(1.33–3.03)	0.001
**High triglyceride level**	2.22	(1.60–3.09)	<0.001	1.69	(1.18–2.43)	0.005
**SE stratum**						
**Medium or higher (ref.)**	1					
**Low/very low**	1.62	(1.11–2.36)	0.012			
**Household Income**						
**>3 SMLV**	1					
**3 SMLV**	1.15	(0.20–6.51)	0.874			
**2 SMLV**	1.72	(0.62–4.78)	0.298			
**1 SMLV**	1.65	(0.60–4.55)	0.330			
**<1 SMLV**	2.64	(0.92–7.56)	0.071			
**Education level**						
**University (ref.)**	1			1		
**Technical**	0.86	(0.28–2.64)	0.796	0.94	(0.29–3.06)	0.919
**Secondary**	1.74	(0.73–4.17)	0.212	1.41	(0.55–3.62)	0.470
**Primary**	4.58	(1.88–11.15)	0.001	2.23	(0.87–5.74)	0.096
**None**	7.38	(2.50–21.82)	<0.001	3.38	(1.07–10.70)	0.038
**Healthcare regimen**						
**Contributive**	1					
**Subsidized**	1.28	(0.89–1.83)	0.174			
**Uninsured/unknown**	0.33	(0.09–1.19)	0.091			

*Source*: authors.

*Note*: SMLV, monthly minimum wage; CI, confidence interval; PR, prevalence ratio; SE, socioeconomic.

The unadjusted prevalence ratios and APRs of metabolic syndrome (ATP III criteria) were higher in females, individuals with unknown or no affiliation with the healthcare regimen, and those with a primary education level (Table 4).

**Table 4:** Variables associated with the prevalence of type 2 diabetes in Bogota, 2022–2023: crude and adjusted regression models.

	ATP III				ALAD			
VARIABLES	UNADJUSTED		ADJUSTED		UNADJUSTED		ADJUSTED	
	PR	95% CI	PR	95% CI	PR	95% CI	PR	95% CI
**Female**	3.19	(2.44–4.16)	3,30	(2.49–4.36)	2.14	(1.62–2.83)	2.29	(1.71–3.08)
**Age, years**								
**<45 (ref.)**	1		1		1		1	
**45–54.9**	1.26	(0.88–1.80)	1.43	(0.99–2.06)	1.02	(0.66–1.57)	1.04	(0.65–1.67)
**55–64.9**	0.89	(0.64–1.25)	1.08	(0.75–1.56)	1.05	(0.71–1.57)	1.13	(0.74–1.73)
**≥65**	1.32	(0.89–1.97)	1.16	(0.80–1.69)	1.65	(1.16–2.35)	1.43	(0.98–2.10)
**SE stratum**								
**Medium or higher (ref.)**	1				1			
**Low/very low**	1.14	(0.80–1.62)			1.26	(0.95–1.67)		
**Household income**								
**>3 SMLV**	1				1			
**3 SMLV**	0.51	(0.17–1.48)			0.80	(0.19–3.42)		
**2 SMLV**	0.45	(0.18–1.14)			1.97	(0.88–4.42)		
**1 SMLV**	0.85	(0.41–1.76)			3.33	(1.42–7.78)		
**<1 SMLV**	0.79	(0.34–1.85)			2.77	(1.16–6.59)		
**Education level**								
**University (ref.)**	1		1		1		1	
**Technical**	0.38	(0.22–0.66)	0.45	(0.26–0.80)	0.64	(0.38–1.08)	0.69	(0.39–1.24)
**Secondary**	0.89	(0.62–1.27)	1.00	(0.69–1.46)	1.20	(0.79–1.82)	1.27	(0.84–1.91)
**Primary**	1.35	(0.90–2.03)	1.61	(1.16–2.25)	2.04	(1.41–2.94)	2.02	(1.38–2.95)
**None**	1.11	(0.54–2.28)	1.13	(0.52–2.47)	1.34	(0.62–2.90)	1.07	(0.46–2.52)
**Healthcare regimen**								
**Contributive**	1		1		1		1	
**Subsidized**	0.86	(0.65–1.13)	0.79	(0.60–1.02)	1.11	(0.85–1.45)	0.94	(0.71–1.24)
**Uninsured/unknown**	1.99	(1.37–2.89)	2.19	(1.48–3.23)	1.43	(0.72–2.84)	1.50	(0.83–2.71)

*Source*: authors.

*Note*: SMLV, monthly minimum wage; CI, confidence interval; ATP III, Adult Treatment Panel III (14); ALAD, Latin American Diabetes Association (17); PR, prevalence ratio; SE, socioeconomic.

Crude and adjusted prevalence ratios of metabolic syndrome in associated variables, according to the ATP III and ALAD criteria, in Bogota, 2022–2023.

When the ALAD criteria were used to define metabolic syndrome, the prevalence ratio of metabolic syndrome was higher in females and individuals with a primary education level (Table 4). The prevalence ratio in individuals who received less than the minimum wage monthly and those aged ≥65 years was higher only in the unadjusted prevalence ratio (Table 4).

## Discussion

The present study is one of the largest field studies conducted in Colombia, with the aim of establishing the prevalence of type 2 diabetes, including the completion of 2,860 household surveys and collection of 1,071 laboratory samples. Likewise, because of the high number of analyzed laboratory samples, this study accurately approximates the real prevalence of metabolic syndrome in the city of Bogotá. This study involved surveys, anthropometric measurements, and laboratory tests in all 19 localities that make up the city of Bogotá.

First, it is highlighted that the prevalence of diabetes found in the population aged ≥18 years reaches 11%. Similar to previous studies, this study considered self‑reporting and the determination of the HbA1C level as sources for the diagnosis of the disease [15, 33, 34].

The previously reported prevalence rates of diabetes for the country ranged from 7.1% [35], according to the International Diabetes Federation, and 8.5%, according to the World Health Organization [36], with an “updated” prevalence of 9.9% estimated for Colombia [4].

A self‑report of 7.5% for type 2 diabetes was found in the surveyed population. The estimated prevalence of diagnosed type 2 diabetes by laboratories and self‑report was 11.0% (95% CI, 9.0–13.5%) in this study.

The prevalence of type 2 diabetes in the city of Bogotá was higher than those reported in previous analyses; thus, 11 of every 100 people in the population aged ≥18 years in Bogotá during the 2022–2023 period had type 2 diabetes. This prevalence is starting to approach that of other Latin American capital cities such as Mexico City or Sao Paulo [4].

In this study, the prevalence rates of metabolic syndrome were 29% and 33% according to the ALAD and ATP III criteria, respectively. Despite both the ALAD and ATP III using the same diagnostic criteria, the difference between the two lies in the fact that for the ALAD, the presence of abdominal obesity is a *conditio sine qua non* for diagnosing the syndrome, whereas for the ATP III, the presence of any three of the five defining features of metabolic syndrome constitutes the diagnostic criteria [20, 23]. The reported prevalence of metabolic syndrome in this study is similar to that accepted by the consensus of the ALAD, which in 2010 recognized a global prevalence in the region of 32%, with a greater predominance in urban areas and females [4].

At least two epidemiological factors could account for the increase in diabetes prevalence compared with previous reports. First, diabetes has a higher prevalence in urban environments than in rural areas, likely due to lower physical activity in the city (longer daily commute times, more time spent in sedentary work, and greater consumption of ultraprocessed foods), and second, there is rapid population growth related to internal and external migration [15, 34–36].

## Limitations

Despite one of the study’s great strengths being the sampling technique that allowed for representativeness across all localities of Bogotá, its development has some limitations, specifically in the fieldwork. These limitations were related to difficulties accessing certain areas of the city because of security reasons or transportation issues, which necessitated extending the data collection times. Additionally, challenges were noted in the collection of blood samples (during the second visit), as well as difficulties in surveying individuals of higher socioeconomic status, as has been referenced in previous cross‑sectional studies [31, 34]. Moreover, the higher number of women in households during the daily survey times led to females and individuals aged 50–69 years being predominant in the surveyed population.

## Conclusions

The high prevalence of diabetes in the city of Bogotá (11%) should lead to the implementation of comprehensive treatment programs for the disease, involving trained medical personnel, endocrinologists, nursing, nutrition, and sports medicine, as well as specialties focused on addressing complications, such as nephrology, ophthalmology, neurology, vascular surgery, and rehabilitation medicine, as it has been suggested by other authors and guidelines. Likewise, significant efforts are needed to reduce the impact of complications related to atherosclerotic diseases (myocardial infarction, stroke, and peripheral arterial disease) [37].

This study provides an approximation of risk factors and socioeconomic conditions related to type 2 diabetes and metabolic syndrome, as previously published [38]. In our study, the factors that were significantly associated with the prevalence of type 2 diabetes in individuals aged ≥18 years in Bogotá were low education level, abdominal obesity, low and very low socioeconomic status, overweight and obesity, high triglyceride level, and older age, specifically those aged ≥55 years. This study has become a new baseline for understanding the problem of diabetes and metabolic syndrome in Bogotá, Colombia, which is a Latin American megacity. Therefore, further in‑depth and follow‑up studies are recommended.

For future research, we recommend conducting a cohort study to monitor patients with metabolic syndrome and assess their risk of developing diabetes in the coming years within this megacity.

## Recommendations and Policy Implications

“The results of this study should raise alarm bells for the city’s health authorities, as a very rapid increase in diabetes mellitus is evident, along with accompanying conditions, such as overweight, obesity and metabolic syndrome, all of which are associated with a higher risk of atherosclerotic cardiovascular disease.

In addition to obvious interventions, such as strengthening the health system to care for and prevent chronic complications in those affected, the implementation of a strong and systematic education strategy at all levels of the population regarding the promotion of healthy eating habits and lifestyle is essential.

We believe that the basis of this strategy should focus primarily on early childhood and schoolchildren who, from the first years of school, should be motivated to follow healthy lifestyle habits, eating habits, physical activity, and movement.

## Prior Presentation

The results of this study were shared in a meeting with Novo Nordisk and the District Health Secretary of Bogotá.

## Additional Files

The additional files for this article can be found as follows:

Supplementary File 1. Table S1. Demographic characteristics of the included individuals.

Supplementary File 2. Table S2. Characteristics of households in the 19 localities of the sample in Bogotá, D.C., 2022–2023.

Supplementary File 3. Table S3. Occupational characteristics of the individuals in the household study in the 19 localities of the sample in Bogotá, D.C., 2022–2023.
