# Supplementary material for: Prevalence of Type 2 Diabetes, Overweight, Obesity, and Metabolic Syndrome in Adults in Bogotá, Colombia, 2022–2023: A Cross‑Sectional Population Survey
Source: Ann Glob Health. 2024 Nov 11;90(1):67. doi: 10.5334/aogh.4539 (PMC11568804; doi:10.5334/aogh.4539)
Supplement: Supplementary File 1. — Table S1. Demographic characteristics of the included individuals. [file agh-90-1-4539-s1.pdf]

Supplemental Table S1.

Variable	Frequency	Percentage
Biological sex		
Female	1,856	65.3%
Male	988	34.7%
Age groups		
<30	454	16.1%
30–39	418	14.8%
40–49	387	13.7%
50–59	556	19.7%
60–69	588	20.8%
70–79	306	10.8%
≥80	116	4.1%
Highest education level attained		
None	64	2.2%
Preschool	6	0.2%
Incomplete primary	277	9.7%
Complete primary	438	15.3%
Incomplete secondary	237	8.3%
Complete secondary	201	7.0%
Incomplete high school	62	2.2%
Complete high school	732	25.6%
Incomplete technical or technological	34	1.2%
Complete technical or technological	378	13.2%
Incomplete university	121	4.2%
Complete university	241	8.4%
Incomplete postgraduate	12	0.4%
Complete postgraduate	55	1.9%
Ethnic group		
Other different	2,755	96.7%
Indigenous	50	1.8%
Black people, Mulatto (Afro-descendant)	40	1.4%
Palenquero	2	0.1%
Raizal from the archipelago	2	0.1%
Gypsy (Rrom)	1	0.0%
What activity occupied most of your time last week?		
Working	1,208	42.6%

Household chores	1,137	40.1%
Other activity	200	7.1%
Looking for work	144	5.1%
Unable to work due to incapacity	77	2.7%
Studying	70	2.5%
Currently employed		
Yes	1,374	48.2%
No	1,477	51.8%
Type of contract		
Verbal	461	58.1%
Written	332	41.9%
Fixed household income		
Less than the minimum wage, <COP$1.000.000	676	23.9%
Equal to the minimum wage, COP$1.000.000	1,398	49.4%
Two minimum wages, ≤COP$2.000.000	565	20.0%
Three minimum wages, ≤COP$3.000.000	104	3.7%
More than three minimum wages, >COP$3,000,000	89	3.1%

Demographic characteristics of the included individuals

Source: authors. COP: Colombian pesos; Exchange rate annual average per United States dollar (USD), 2022: COP$4,256; COP$1.000.000 = USD 234,45; COP$2.000.000 = USD $469,90; COP$3.000.000 = USD $704,90
