# Supplementary material for: Prevalence of Type 2 Diabetes, Overweight, Obesity, and Metabolic Syndrome in Adults in Bogotá, Colombia, 2022–2023: A Cross‑Sectional Population Survey
Source: Ann Glob Health. 2024 Nov 11;90(1):67. doi: 10.5334/aogh.4539 (PMC11568804; doi:10.5334/aogh.4539)
Supplement: Supplementary File 2. — Table S2. Characteristics of households in the 19 localities of the sample in Bogotá, D.C., 2022–2023. [file agh-90-1-4539-s2.pdf]

Supplemental Table S2.

Variable	Frequency	Percentage
Type of housing		
House	1,959	68.6%
Apartment	770	27.0%
Room(s) in tenement	84	2.9%
Room(s) in other type of structure	35	1.2%
Other	9	0.3%
Socioeconomic stratum		
Very low	414	15.4%
Low	1,381	51.4%
Medium low	814	30.3%
Medium	77	2.9%
Medium high	3	0.1%
Services accounted for in the household		
Electricity		
No	67	2.3%
Yes	2,793	97.7%
Natural gas connected to the public network		
No	156	5.5%
Yes	2,704	94.5%
Sewerage		
No	179	6.3%
Yes	2,681	93.7%
Garbage collection		
No	107	3.7%
Yes	2,753	96.3%
Water supply		
No	115	4.0%
Yes	2,745	96.0%
Fixed telephone		
No	1,425	49.8%
Yes	1,435	50.2%
Prefer not to say		
No	2,662	93.1%
Yes	198	6.9%
Means to dispose of garbage and waste in their household		

Public or private collection	2,839	99.6%
Disposed of in another way	8	0.3%
Discharged into a river, stream, canal, or lake	4	0.1%
Places where people in this household prepare their food		
In a room used only for cooking	2,594	91.0%
In a dining room with a sink	183	6.4%
In a room used for sleeping as well	47	1.6%
Nowhere, they don't prepare food	12	0.4%
In a courtyard, hallway, arbor, or outdoors	9	0.3%
In a dining room without a sink	5	0.2%
Type of housing occupied by this household		
Renting or subletting	1,445	50.8%
Owned, fully paid	1,092	38.4%
Cooperative property	155	5.5%
Owned, still paying for it	87	3.1%
Other	54	1.9%
In usufruct	7	0.2%
Possession without title (de facto occupant)	4	0.1%

Characteristics of households in the 19 localities of the sample in Bogotá, D.C., 2022–2023

Source: authors.
