# Supplementary material for: Prevalence of Type 2 Diabetes, Overweight, Obesity, and Metabolic Syndrome in Adults in Bogotá, Colombia, 2022–2023: A Cross‑Sectional Population Survey
Source: Ann Glob Health. 2024 Nov 11;90(1):67. doi: 10.5334/aogh.4539 (PMC11568804; doi:10.5334/aogh.4539)
Supplement: Supplementary File 3. — Table S3. Occupational characteristics of the individuals in the household study in the 19 localities of the sample in Bogotá, D.C., 2022–2023. [file agh-90-1-4539-s3.pdf]

Supplemental Table S3.

Occupational variables		Frequency	Percentage
Current pension fund contribution	Yes	558	19.5%
	No	2,046	71.7%
	Already retired	251	8.8%
Family compensation fund	Yes	518	18.2%
	No	2,288	80.4%
	Not sure, not informed	40	1.4%
Compatibility of work schedule and family responsibilities	Yes	1,104	80.5%
	No	267	19.5%
Main means of transportation to get to your workplace	Does not travel	375	27.4%
	On foot	373	27.2%
	Articulated transport (Transmilenio and others)	212	15.5%
	Bicycle	131	9.6%
	Motorcycle	77	5.6%
	Private car	52	3.8%
	Urban bus	46	3.4%
	Intermunicipal bus	36	2.6%
	Other	24	1.8%
	Company transportation	20	1.5%
	Taxi	12	0.9%
	Motorcycle taxi	10	0.7%
	Horse	2	0.1%
Performing tasks in other households or institutions	Yes	159	5.6%
	No	2,701	94.4%
Taking care of or attending to children	Yes	310	10.8%
	No	2,550	89.2%
Taking care of sick, elderly, and/or disabled people	Yes	157	5.5%
	No	2,703	94.5%
Attending courses or training events	Yes	79	2.8%
	No	2,781	97.2%
Other	Yes	1,089	38.1%
	No	1,771	61.9%

Occupational characteristics of the individuals in the household study, in the 19 localities of the sample in Bogotá, D.C., 2022–2023

Source: authors.
